# 'We call it the shaking illness': perceptions and experiences of Parkinson's disease in rural northern Tanzania

**DOI:** 10.1186/1471-2458-11-219

**Published:** 2011-04-08

**Authors:** Gerry Mshana, Catherine L Dotchin, Richard W Walker

**Affiliations:** 1 National Institute for Medical Research, P.O. Box 1462, Mwanza, Tanzania; 2 Institute for Ageing and Health, Newcastle University, UK; 3 North Tyneside General Hospital, Rake Lane, North Shields, Newcastle, UK

## Abstract

**Background:**

Parkinson disease (PD) causes physical disability that negatively affects the quality of life of the sufferer's and their families. There are no Parkinson's disease (PD) social science studies published from Africa. This paper presents findings from a qualitative research study on how PD is perceived and treated in a population of approximately 161,000 within a demographic surveillance site in rural Tanzania.

**Methods:**

We conducted in-depth interviews with 28 PD sufferers, 28 carers, 4 health workers and 2 traditional healers. In addition, 6 focus group discussions were conducted in 3 villages to investigate wider community views of PD.

**Results:**

PD sufferers expressed frustration with the physical, psychological, social and economic consequences of the illness. Feelings of a diminished quality of life characterised by dependency, stigma and social isolation were common. Additionally, a handful of male sufferers related their sexual incompetence to the illness. Carers complained of lost income opportunities and social isolation resulting from caring for sufferers. Misconceptions about the cause, symptoms and appropriated PD treatment were widespread. Only 2 PD sufferers had commenced western type treatment through outsourcing drugs from other parts of the country and outside of Tanzania.

**Conclusions:**

This study highlights the urgent need for PD awareness and treatment interventions in such settings. Such interventions need to address the concerns and needs of sufferers, their carers and the wider community, including the health care system.

## Background

Generally, there have been few studies of neurological illnesses conducted in Africa leading to concerns that this area has been neglected [1]. Estimates of the prevalence for Parkinson's disease (PD) worldwide have ranged from 10 to 405 per 100,000 of the population [2]. Reasons for the wide variation may include differences in case-finding procedures, in the diagnostic criteria applied, and in the response rate of the populations studied, notwithstanding 'real' differences due to genetic and/or environmental factors. It has been reported that black African populations have the lowest prevalence of PD, but it is not clear whether this reflects a true difference in susceptibility in these populations [3]. Clinical and epidemiological PD studies conducted in sub-Saharan Africa (SSA) have reported lower prevalence rates than those from Europe and North America [4,5].

We conducted a community-based prevalence study of Parkinson's disease in rural northern Tanzania [5]. In a population of approximately 161,000 people living in a demographic surveillance site, 32 cases of PD were detected; 23 were male. Age-standardised prevalence rates to the UK population for men, women and combined were 64/100,000, 20/100,000 and 40/100,000 respectively. The majority of cases were undiagnosed and untreated prior to the study. The figures for men were similar to previous reports for African Americans in Manhatten [6] and an Indian population [7], but significantly lower than prevalence estimates from developed countries.

To our knowledge there are no published social science PD studies conducted in Africa despite calls for more detailed social studies on non-communicable illnesses on the continent [8]. The aims of this study were to investigate the experience and treatment seeking behaviours of PD sufferers and their carers together with community understandings of PD in a rural part of Tanzania.

In carrying out the study, we employed the sociology of chronic illness approach. In health related research, the approach has mainly been used to explore the social aspects of illnesses enquiring about the meaning and consequences of an illness to sufferers and their carers [9]. It has been demonstrated that making sense of illness and suffering is an important endeavour for sufferers of chronic illnesses. For example, Nettleton [10] reports that people with medically unexplained symptoms (MUS) struggle to ascertain their source of suffering. When they fail to locate the source, they express shame and guilt which negatively affects their sense of self and social identity. Furthermore, sufferers may convey a sense of 'loss of self' as certain 'taken for granted' body images and functions are altered [11]. In most cases, the alteration of the 'self' resulting from suffering from a chronic illness leads to sufferers living a restricted, isolated, life feeling they are disgraced and posing a burden to their families. Chronic illnesses may also lead to a series of outcomes such as loss of productive function, financial difficulties, family strain and stigma [11].

The onset and development of a chronic, progressive and disabling illness such as PD could have a major impact on the quality of life of the affected person and their family [12]. News of a PD diagnosis may be shocking, distressing and disappointing. During the course of their illness, from first symptoms to severe disability, PD sufferers may repeatedly experience uncertainty and pose many questions about their condition [13]. PD sufferers participating in one study reported experiencing a loss of physical and mental functioning, independence and self-identity and were afraid of further losses [14]. PD may therefore lead to a generally lower quality of life for sufferers, more so for those living in rural areas. For example, in Croatia, PD sufferers living in rural areas had a lower quality of life score than urban sufferers when examined using the quality of life (PDQ-39) questionnaire [15]. This may be partly because people in urban areas have better access to healthcare and a better life in general [15]. On the other hand, carers may also face challenges and changes in their lives. They may suffer from loss of productivity and frustration from their caring experience.

A variety of studies have reported widespread PD stigma. In some instances, PD has been described as 'a problem of shame' [16]. The shame may due to various reasons such as not being able to speak 'normally' and the resulting physical dependency. In the UK, where more people are aware of the illness, PD stigma is more directed at younger sufferers than older sufferers [17]. PD is more 'visible' in younger sufferers and seen as 'appropriate' with the age of older sufferers [17] as it may be perceived as part of the ageing process [18]. In Croatia, PD patients living in rural areas felt that they were more stigmatised compared to their urban counterparts [15]. PD related stigma may result in social isolation as sufferers 'retreat from the public world' [16]. In a study conducted in the UK, swallowing difficulties led to PD sufferers going through social isolation as they could not participate in eating and drinking which are important social functions [19].

PD may also affect the sexual lives of sufferers. A study found that women with PD were less satisfied with their sexual experience compared to those without the illness [20]. This is a matter of perception or only a problem of older PD sufferers as another study found that younger PD sufferers (mean age of 45) had no difference in sexual function when compared with healthy counterparts [21].

Coping with PD may vary from community to community. PD sufferers may receive support from care institutions and hospitals which when combined with social support from their families and friends, could lead to better quality of life outcomes [22]. For example, Bingham and Habermann [23] reported that PD sufferers and their families managed the illness through religious belief and faith, purpose and meaning, and the support of family and friends. The changing landscape in the medical management of chronic illnesses and healthcare mean that the role of sufferers in the management of their conditions is increasing. As self management has become increasingly common, it is even more important to understand and document the lived experiences of the sufferers [24].

Kaufman [25] points out that 'knowing and conveying the experience of the other depends upon interpretation', and the process of interpretation entails considering the context upon which the experience is based. Therefore, in addition to PD sufferers and carers, we included in our sample other groups of people from the study area in order to get a broader view of how PD is framed and treated in rural Hai. Insights from these groups were helpful in interpreting what the sufferers and carers experience within the wider community's context.

## Methods

### Study site and population

We conducted the study within the Adult Morbidity and Mortality Project (AMMP) area of the Hai district with a population of 161,162. The Chagga comprise the largest ethnic group in the district (75%) followed by the Maasai (12%), and Pare (10%) while other ethnic groups make up the remaining 3%. The majority of the population are Christians (79%) while the remaining 21% are Muslims [26]. The district has two main geographic features; the mountainous terrain towards Mount Kilimanjaro and the flat lowlands plains on the southern part of the mountain. The fertile mountainous terrain has the highest population density. Agriculture is the main economic activity in Hai. Residents in the lowlands produce seasonal crops such as maize, cassava, sorghum, beans, groundnuts and sunflower. The highlands, on the mountain slopes, receive more rain and are composed of fertile volcanic soil. Bananas, beans and coffee are the main agricultural produce in this part.

### Study participants and procedures

We employed two qualitative research methods for data collection, semi structured interviews (SSIs) and focus group discussions (FGDs). We conducted a total of 62 SSIs with a purposively selected sample of the following groups of people:

#### PD sufferers (n = 28)

PD sufferers were identified and recruited through the community based PD prevalence study [5]. For the study we carried out a house-to-house survey in June and July 2005 with 6 questions to identify people who may potentially have PD. The questions covered tremor, bradykinesia and postural instability. The fourth cardinal feature of PD, rigidity was not covered, as previous studies have indicated this does not increase the sensitivity or specificity of screening tools. Those who replied positively to any of the questions were invited to take part in the prevalence study. Other case finding methods were utilized as detailed [5]. They then underwent a structured history and examination by the research doctor to determine whether or not they had PD according to the UK Parkinson's Disease Society Brain Bank Criteria [27]. We had planned to interview 29 PD sufferers but one female sufferer was excluded due to cognitive impairment.

#### Carers of the PD sufferers (n = 28)

We interviewed one carer for each patient. We identified the carers through asking the sufferers who was the main person caring for them. During home visits, the research team observed household dynamics to confirm who among the household members seemed to be close to the sufferer.

#### Health Workers (n = 4)

These were clinical officers selected with the help of study community coordinators from 4 health clinics in the study area.

#### Traditional healers (n = 2)

These were picked from information obtained from study participants and study community coordinators. We had planned to interview 4 traditional healers, but had to exclude two after they informed us that they had never heard of the illness nor had they treated someone suffering from it.

We conducted 6 FGDs (3 males, 3 females) in three villages. The number of participants for each FGD ranged between 7-10. In total we had 50 FGDs participants (26 females, 24 males). FGDs participants were recruited purposively from people with different socio-economic backgrounds to ensure a representation of different groups and views. We invited the participants to participate in the FGDs through a network of AMMP community enumerators who reside in the villages.

The SSIs and FGDs were conducted in Swahili (the national language) by same sex researchers to give the participants the confidence of expressing themselves. SSIs and FGD guides had questions on participants' and community understanding of PD, their experiences as sufferers or carers, coping mechanisms and treatment seeking behaviours. The SSIs and FGDs were tape recorded after permission was obtained from participants. Participants were given verbal and written information about the purpose and process of the study. During the introduction research staff emphasized that participation was entirely voluntary and no one was obliged to answer the questions. Confidentiality and anonymity were ensured and a signed or thumb print consent was given by all participants. Ethical approval for the study was obtained from the Medical Research Coordination Committee (MRCC) of the Tanzanian National Institute for Medical Research.

### Data analysis

SSIs and FGDs tapes were transcribed verbatim. Data was analysed by two researchers in three stages using an inductive approach [28]. In the first instance, a trained research assistant listened to all the audiotapes and summarised the data according to emerging themes. Secondly, a senior social scientist reviewed all data transcripts also summarising emerging themes. The two sets of themes were then compared and discussed by the two analysers. Thereafter, hypotheses were formulated from the emerging patterns and subsequently data revisited to test hyp otheses.

## Results

### Demographics

The age range of PD sufferers was 45-94 years, of whom 26 (93%) were older than 64 years. 32 (52%) of the interview respondents were male and 30 (48%) females. 57 (92%) of the respondents were Christians, while the remaining 5 (8%) were Muslims. 14 (22.5%) had never attended school, 34 (55%) had primary school education and the remaining 14 (22.5%) had education above primary school level (secondary or college). 42 (68%) were subsistence farmers and the remaining 20 (32%) had other occupations such as teaching and small business owners.

### Terminology

The Swahili definition of PD provided by an English to Swahili dictionary is ***ugonjwa wa kutetemeka ***[shaking or tremor disease/illness] and ***ugonjwa wa kukakamaa ***[rigidity disease/illness] [29]. In Hai, the terminology used for PD mainly referred to either the symptoms or perceived causes of the illness. The most common phrase used was ***ugonjwa wa kutetemeka***. Another term used was, ***ugonjwa wa baridi ***[cold illness] which was related to the tremor since someone feeling cold may occasionally shiver. The other term was ***ugonjwa wa uzee ***[old age illness] due to the perception that the illness mainly affects old people. Other terms used to refer to the illness were, ***ugonjwa wa akili ***[psychiatric illness] due to cognitive impairment manifesting in some sufferers, ***ugonjwa wa kupooza ***[paralysis illness] and ***ugonjwa wa mifupa ***[illness of the bones] which were associated with the physical disability resulting from the illness.

### Experience of sufferers

All PD sufferers felt that the illness had changed their lives drastically for the worse. A few (4) sufferers were extremely bitter that the illness had generally made their life hopeless. Most described the illness in a very negative way as demonstrated in phrases such as:

*'This illness is just problems'; 'This illness is like poison'; 'This illness is like something rotting in the body'; 'This illness is very bad, dirty and makes one despair' ; 'This illness has made me stupid and dumb'*.

Most sufferers mentioned physical symptoms and disabilities as the prominent effect of the illness. A 65 year old female sufferer said:

*'I get tired...the tremor is not only on the hands...the whole body tremors...even the intestines tremor...and at night I get tired...I become unhappy'*.

Many expressed frustration with failure to engage in physical activities they were able to do prior to the onset of the illness. PD sufferers said they could no longer: dress themselves (18), walk without assistance (21), eat properly (20) or turn in bed (5). Sufferers also talked of the way the physical shortcomings had led them to live isolated lives. A 79 years old male sufferer said:

*'For us who have this illness, we have noticed that our condition has changed into a condition that is not normal...for example, I used to like to go out and sit somewhere taking alcohol with my friends, but now you cannot go out and sit with your friends because of the leg disability...and also you feel shy...you have no strength'*.

A few (4) male sufferers complained that the illness has made them unable to have sex with their spouses due to erectile dysfunction. Two reported that the illness had caused constant fatigue making them fall asleep frequently.

Sufferers also lamented of dire economic consequences on their lives caused by the illness. Most (23) said it had made them unable to work which had led to a drop in, or total loss of, their income. As a result, the illness has made the sufferers totally dependent on their siblings and relatives. Some (5) reported having experienced PD related stigma from family and community members.

### Experience of carers

The majority of the carers were females, mainly wives of the sufferer's (43%) and daughters or daughters in law (18%). Other carers were employed carers (3), sons (3) and a grandson, father, husband, brother in law and niece (1 for each category). In all three cases of the employed carers, it is the children of the sufferers (living in towns) who send money for their salaries. Interestingly though, none of the three sufferers with employed carers had obtained appropriate PD treatment.

Carers talked about several ways in which caring for PD sufferers had changed their lives. With the exception of the three employed carers, most carers emphasized the economic loss they had experienced. Carers talked of how their duty had led them to fail to fully engage in income generating activities resulting in income reduction (16). For example a 42 year old carer of his parent said:

*'My income has reduced drastically because the little I get is for taking care of the ill person (sufferer)...and the family still depends on you...and as you understand life these days, the cost of running a home is high...therefore the little I was saving so as to take care of the family I have to now split...and even that is not available because I use a lot of time to care for the ill'*.

Carers also talked of psychological humiliation since they had become dependent on their relatives as a result of failing to engage in income generating activities such as farming. A few (3) talked about how the caring task had resulted in social isolation as they could not go away from home to visit friends and relatives or attend church and funerals. In addition, some of the carers talked of the hopelessness they experienced when the people they cared for developed PD symptoms as they did not know exactly what the illness was and how to help them. Many said they initially thought the illness was a result of old age.

They recounted that though they took the sufferers to hospital, they had not seen any improvement in their condition and therefore felt helpless. The carers' experiences were expressed in phrases such as: *'Caring for the sufferer has made my life very difficult'; 'Caring for the sufferer is a big burden to me'; 'This illness has made us become very poor'*.

### Awareness

Generally, PD awareness is very low in Hai and very few people had ever heard of the illness. Most respondents said they did not know what causes PD and could only guess. Many thought it was caused by cold weather and witchcraft. PD sufferers said that they were not aware that they had the illness until after they were visited by the PD prevalence study staff. Prior to that, they thought they had other forms of illnesses, such as bone related illnesses, as manifested by the symptoms they were experiencing. A few thought they had either been bewitched or were suffering from a form of a curse and opted to consult traditional healers for treatment. A 41 year old male sufferer said:

*'I consulted a traditional healer a few times...not many times...like three times...he made incisions on my body and applied medicine...I did not get any relief...the pain remained as before...but later I decided to stop and continue with hospital treatment'*.

The two youngest sufferers (aged 41 and 57) questioned more than others the cause of their illness. They expressed a strong opinion that they might have been bewitched or be suffering from a curse. This echoed a common belief in Hai that people get '*the shaking illness*' through witchcraft after taking forcefully something belonging to other people [***dhulumu***]. However, when probed by researchers, the two young sufferers could not recall an instance when they had done such a thing.

Such misunderstandings about the possible causes of PD are widespread among different groups of people, including the traditional healers and health workers. Interviewed health workers mentioned stress, hypertension and poisoning as the possible cause of PD. The 2 traditional healers expressed similar PD views as other community members. They could not give a specific name for the illness but talked of it through describing the major symptoms such as the tremor and walking difficulties. Their treatment regime comprised of undoing the witchcraft and treating the visible symptoms.

Despite the outlined misconceptions, some respondents had correct notions about PD, such as that it mainly affects the elderly. After probing some respondents from all groups mentioned correct PD symptoms such as tremor, walking difficulties, body pain and dysphasia. Tables 1 and 2 illustrate findings from the different participating groups:

**Table 1:** PD causes, symptoms and prevention measures mentioned by respondents *

	Cause	Symptoms	Prevention
**Mentioned by sufferers and carers**	Cold weather (10) Old age (8) Thinking deeply (3) High blood pressure (3) Alcohol (6) Lack of vitamins (4) Use of hospital medicine (3) Use of insecticides (1) Curse (4) Witchcraft (8) Doing lots of work (1) Genetic inheritance (1) Having sex during menses (1) Pregnant mother eating taboo foods (1) Don't know (17)	Leg and arm tremor (38) Failing to walk (24) Failing to do manual work (18) Body weakness (11) Leg pains (12) Leg numbness (7) Falling without any reason (7) Feeling hotness in legs (4) Being forgetful (4) Failing to have sex (4) Abdominal gas (2) Sweating heavily (2) Body paralysis (1) Cognitive impairment (1) Hoarse voice (1) Walking while bending (1) Neck stiffness (1)	Vaccination (2) Early treatment (1) Good nutrition (1) Refraining from taking alcohol (1) Stop thinking deeply (1) Hygiene practices such as washing hands before eating (1) Don't know (49)

**Mentioned by health workers**	Alcohol (3) Age (2) Head injury (2) Viral encephalitis (1) Brain degenerative disease (2) Chemicals such as carbon monoxide and mercury (1) Hypertension (1) Thinking deeply (1) Genetic inheritance (1) Poisoning (1)	Leg and arm tremor (4) Walking slowly (2) Failing to walk (2) Dysphasia (2) Leg pains (1) Tightening of leg muscles (1) Lack of energy (2)	Refraining from taking alcohol (3) Seeking medical help early (2) Physical exercise (2) Vaccination (1) Avoid contact with chemicals (1) Don't know (1)

*Numbers in columns may not add up to 100% as some respondents mentioned more than one item

**Table 2:** PD causes, symptoms and prevention measures mentioned by FGDs participants

Cause	Symptoms	Prevention
Thinking deeply Alcohol Not eating food Witchcraft Curses Head injury Poisoning from farm produce, industry and traditional medicine Cold Smoking marijuana and other drug abuse AIDS Malaria High blood pressure	Leg and arm tremor Failing to work Failing to walk Cognitive impairment Dysphasia Losing energy Walking short steps Walking while bending Body numbness	Stop thinking deeply Early treatment Refraining from using alcohol Stop using medicines without proper direction Stop using foodstuffs with chemicals Stop hitting children on heads

### Treatment seeking

Most PD sufferers were not aware that they were suffering from PD and therefore had not received appropriate treatment [5]. Most had sought treatment for the specific symptoms they were experiencing such as the tremor, cognitive impairment and walking difficulties.

When we carried out the study, only 2 sufferers were on western type PD treatment. These sufferers had been diagnosed in hospital and were receiving support from their children and relatives to purchase the drugs from outside of Hai district. This is a necessary endeavour as PD drugs are not stocked in the public or private health facilities or in local pharmacies. One sufferer was buying the drugs from a nearby town (Arusha) and the other was sourcing them through a relative residing in Nairobi, Kenya. Both complained of the high costs for the drugs as a month's supply would cost 40,000/- Tanzanian shillings [equivalent to about 33 US dollars]. Due to the high cost, the two sufferers said they sometimes run out of money to order the drugs causing a return of their symptoms. The two sufferers reported getting great relief after using the drugs as they were able to go about their daily activities as normal. Some of the other PD sufferers sought healing services from traditional or faith healers. Faith healing was done through being prayed for by church members, either in their homes, in church or at public meetings. Churches involved in faith healing were the Lutheran, Catholic and Pentecostal churches.

Treatment decisions were reached either solely by the sufferer themselves, or in collaboration with family members. In some instances, and depending on the nature of the social networks of the sufferer and family, neighbours and family friends could be consulted. The primary consideration in treatment seeking decisions was the ability of the sufferer or family to afford the treatment. Affordability involved costs for transport to and from hospital, paying for consultations and drugs. Some sufferers said that they had not been going to hospital as often as they would like because family members regard their condition as 'normal' with old age.

## Discussion

This is a pioneering study on the social aspects of PD in Africa. We realise that our study could have benefited more from longitudinal ethnographic data exploring further the experiences and meaning of PD to the general life of sufferers and their carers within the community context. We are planning to carry out such follow-up studies in the near future.

From this study, it is evident that PD awareness is very low in this rural part of Tanzania. This is despite the fact that all the sufferers (with their families) had been seen as part of a prevalence study, through which they will have gained information about PD. The majority of respondents (including the sufferers and their carers) were not aware of the symptoms and appropriate treatment for PD. In Hai, PD is generally recognised and treated after the manifestation of symptoms such as tremor, cognitive impairment and walking difficulties. The diversity of the perceived causes of PD and its symptoms further highlight unfamiliarity with the illness. Misconceptions about the illness were widespread among all participating groups. This highlights the need for PD awareness interventions to address issues such as the belief that PD is caused by witchcraft. Since such beliefs have also been documented in relation to other non-communicable illnesses e.g. stroke [30] a wider awareness campaign targeting a series of non-communicable illnesses may be cost effective. Such an effort is warranted although we realise that infectious diseases, such as malaria and AIDS, still comprise the bulk of disease burden in sub-Saharan Africa.

In rural Hai, physical, psychological, economic and social suffering framed the experience of PD sufferers. Failure to fend for themselves and their families and generate income made sufferers express a sense of 'loss of self' as highlighted in studies elsewhere [9,11]. In this context, the definition of 'the self' is extended to include obligations and duties to the sufferer's dependants. PD sufferers do not gauge the effects of the illness only to themselves, but include the other aspect of 'their self' such as spouses and other family members.

Physical disabilities resulting from PD make sufferers lead a reliant and socially isolated life as also reported by other studies [16,19]. The problems are not restricted to motor symptoms [31]. This situation may be further worsened by PD related stigma. Misconceptions about the cause of PD, such as witchcraft associated with taking things forcefully from other people (***dhulumu***), exacerbate the stigma associated with PD. In addition, the perception that PD is a 'normal' old age illness may lead to sufferers' not getting timely and proper care. Inability to secure proper care is further jeopardised by the unavailability of PD drugs in local health facilities and pharmacies. As a result, PD sufferers are unlikely to get appropriate treatment even if diagnosed, making their situation even more challenging [32].

The overall quality of life for sufferers including marriage could additionally deteriorate with reports of erectile dysfunction by some male sufferers. Although we did not systematically investigate this in our study, reports of similar findings on sexual dissatisfaction elsewhere [20] despite contradictory findings with younger sufferers [21], highlight the need to further explore this issue in order to improve the quality of life of sufferers.

Interventions, such as self support groups, could prove useful in providing social support for PD sufferers in this locality. Involvement of health professionals in such endeavours may be crucial in order to ensure that sufferers find answers to some of their questions on possible causes of the illness. In developed countries, the support of self help groups is seen as an important intervention for people with chronic illnesses [14]. In some countries, there have been a series of innovative interventions for people with chronic illnesses such as the Expert Patients Programme (EPP) [33]. However, there is debate on whether these interventions should be lay (sufferers) or expert (health professionals) led, to achieve maximum benefit [33]. Nonetheless, it is generally acknowledged that self management programmes are beneficial to chronic illnesses sufferers [14,22,33].

The majority of PD carers in our study area were family members, mainly women. However, this observation must be treated with caution as our sampling procedure was not random. One reason that the majority of carers were women is that most were spouses and the majority of patients were male. Despite this limitation, such an observation calls for any care and support intervention to prioritise the needs of specific caring groups such as women. Our study has shown that carers also suffer economic and social consequences as a result of their caring responsibilities. Reports of carers failing to engage in social activities such as funerals and festivals highlight the serious social consequences of caring for a PD sufferer. This further demonstrates that the consequences of PD extend beyond the sufferers especially in this context where help from health professionals such a community nurses may not be readily available.

Most of the PD sufferers we saw had not obtained any western style treatment. This may be a manifestation of either lack of proper diagnosis due to the lack of capacity within the healthcare system, or inability of sufferers to 'outsource' the drugs. The reported lost opportunity to generate income may make it difficult for sufferers and their carers to afford treatment even if it were available within the existing healthcare system. Additionally, as health workers were unclear about PD, it is imperative for any designed intervention to prioritise training of health workers on diagnosis and management of PD. Furthermore, any PD treatment programmes in developing countries settings must ensure that the costs of the lifetime treatment drugs is affordable to resource constrained populations residing in the rural areas. An example of such an approach may involve technical corporation and joint efforts to fund-raise for the treatment programmes between local health authorities (such as the office of the district medical officer) and national and international partners.

## Conclusions

Our study underlines the need for initiating appropriate planning for PD diagnosis and treatment interventions in rural Africa taking into consideration the contextual and healthcare system needs. In resource poor settings, such as sub-Saharan Africa, such interventions must be cost effective and tailored to suit the local context. Additionally, it is important that such interventions ensure a reliable, affordable and sustainable supply of PD drugs. Health policy setting at the national and international levels needs to recognise that early and appropriate intervention of the 'non traditional' illnesses in developing countries, such as PD, may offer a better chance of success.

## Competing interests

The authors declare that they have no competing interests.

## Authors' contributions

GM and RRW designed the study. GM oversaw the data collection and analysis. GM, RRW and CLD contributed in drafting and refining the manuscript. All authors read and approved the final version of the manuscript.

## Pre-publication history

The pre-publication history for this paper can be accessed here:

http://www.biomedcentral.com/1471-2458/11/219/prepub
